# Enzymatic Characterization of a Novel HSL Family IV Esterase EstD04 from *Pseudomonas* sp. D01 in Mealworm Gut Microbiota

**DOI:** 10.3390/molecules28145410

**Published:** 2023-07-14

**Authors:** Jung-En Kuan, Chih-Hsuan Tsai, Chun-Chi Chou, Cindy Wu, Whei-Fen Wu

**Affiliations:** 1 Department of Agricultural Chemistry, College of Bio-Resource and Agriculture, National Taiwan University, Taipei 10617, Taiwan; r06623023@ntu.edu.tw (J.-E.K.); ntukenny@gmail.com (C.-C.C.); cindy-wu@alumini.brown.edu (C.W.); 2 Department of Microbiology and Immunology, College of Medicine, National Cheng Kung University, Tainan 701401, Taiwan

**Keywords:** type IV esterase, *Pseudomonas* sp., bacterial hormone-sensitive lipase (bHSL), mealworm’s gut-microbiota, broad substrate specificity

## Abstract

*Pseudomonas* sp. D01, capable of growing in tributyrin medium, was isolated from the gut microbiota of yellow mealworm. By using in silico analyses, we discovered a hypothesized esterase encoding gene in the D01 bacterium, and its encoded protein, EstD04, was classified as a bacterial hormone-sensitive lipase (bHSL) of the type IV lipase family. The study revealed that the recombinant EstD04-His(6x) protein exhibited esterase activity and broad substrate specificity, as it was capable of hydrolyzing *p*-nitrophenyl derivatives with different acyl chain lengths. By using the most favorable substrate *p*-nitrophenyl butyrate (C_4_), we defined the optimal temperature and pH value for EstD04 esterase activity as 40 °C and pH 8, respectively, with a catalytic efficiency (*k_cat_*/*K_m_*) of 6.17 × 10^3^ mM^−1^ s^−1^ at 40 °C. EstD04 demonstrated high stability between pH 8 and 10, and thus, it might be capably used as an alkaline esterase in industrial applications. The addition of Mg^2+^ and NH_4_^+^, as well as DMSO, could stimulate EstD04 enzyme activity. Based on bioinformatic motif analyses and tertiary structural simulation, we determined EstD04 to be a typical bHSL protein with highly conserved motifs, including a triad catalytic center (Ser^160^, Glu^253^, and His^283^), two cap regions, hinge sites, and an oxyanion hole, which are important for the type IV enzyme activity. Moreover, the sequence analysis suggested that the two unique discrete cap regions of EstD04 may contribute to its alkali mesophilic nature, allowing EstD04 to exhibit extremely distinct physiological properties from its evolutionarily closest esterase.

## 1. Introduction

Esterases and lipases are lipolytic enzymes that are members of the α/β hydrolase family. In general, the former are carboxyl ester hydrolases (EC 3.1.1.1), which hydrolyze triglycerides with short or medium monoester chains (less than 10–12 carbon atoms). The latter are triacylglycerol hydrolases (EC 3.1.1.3), which hydrolyze water-insoluble long-chain triglycerides (more than 10–12 carbon atoms) [1,2,3,4]. These lipolytic enzymes hydrolyze fats and oils and yield free fatty acids, diacylglycerols, monoacylglycerols, and glycerols [5]. In addition to their hydrolytic activities on carboxylic ester bonds, these lipolytic enzymes also perform different catalytic activities on the esterification and transesterification reactions [4,5,6]. Particularly, the lipolytic enzymes are able to act as chemo-, regio-, and stereoselective catalysts to various substrates [4,5,7,8]. Hence, the usage of lipolytic enzymes is beneficial in numerous fields, such as foods, detergents, chemicals, cosmetics, pharmaceuticals, waste treatments, and biofuels [4,9,10]. Due to its wide application, globally, there is an increasing demand for novel lipolytic enzymes.

Primarily isolated from bacteria, the esterases are abundant in nature and more useful due to their broad range of catalytic activities, high yields, and ease of genetic manipulations [4,11]. Recently, some esterases have been isolated from metagenomes [12,13,14,15,16,17,18]. It was reported that bacterial esterases could adopt monomers or oligomers with molecular weights in the range of 25–85 kDa [19]. As they belong to the class of α/β-hydrolases, their active sites generally consist of highly conserved catalytic triad such as a nucleophilic residue (Ser, Cys, or Asp), a catalytic acidic residue (Glu or Asp), and a proton carrier His [20,21] in close spatial proximity. Thus, a short consensus sequence, GXSXG pentapeptide, in which X stands for various amino acids, commonly exists in esterases [11,22,23].

The progressive discovery of new esterases by metagenomic approaches elicited an expansion of the original taxonomy to 35 families, of which 11 were true lipase families [24]. However, lipolytic enzymes from different microbes still harbor diverse amino-acid sequences around the serine active site and possess considerably different characteristics at molecular and biochemical levels [8]. Therefore, lipases have been further classified into seven phylogenetic clusters [8]. Among them, type IV esterases belong to bacterial hormone-sensitive lipases (bHSLs), which have a C-terminal catalytic domain homologous to eukaryotic hormone-sensitive lipases [8,11]. The bHSLs share a similar catalytic triad with eukaryotic HSLs, in which the active site is composed of Ser, Glu/Asp, and His in the conserved [GXSXG, (E/D)XL, and HXF] motifs [11,25,26]. The most salient structural differences between bHSLs in bacteria are located in the cap region of the N-terminal domain, which shields the catalytic center and mediates efficient substrate binding [15,18,27]. In spite of the increasing numbers of newly discovered bHSLs, information on this enzyme family is still limited, and only a few of them have been characterized [8,11].

In this study, we isolated a bacterium *Pseudomonas* sp. D01 from the microbiota of mealworm gut and discovered a novel type IV esterase EstD04 from the bacterium. *Pseudomonas* sp. D01 was able to utilize tributyrin as its sole carbon source and was identified through 16S rDNA phylogenetic analyses. We characterized EstD04 esterase through bioinformatic analyses and molecular biotechniques and cloned and expressed EstD04 in the *Escherichia coli* system. Enzymatic characteristics of purified EstD04 were delineated, including optimal and tolerant temperature and pH and effects of metal ions, cations, organic solvents, or detergents on EstD04 esterase activity. Furthermore, we also identified special conserved motifs in the EstD04 protein through bioinformatic analyses and predicted its tertiary structure. Our results indicated that EstD04 belongs to bHSL family IV and can hydrolyze various substrates. Moreover, its two cap domains with distinct sequences were implied to be important for alkali mesophilic lipolytic activity and significant traits in the evolution of esterases.

## 2. Results

### 2.1. Screening and Identification of Bacterial Strain D01 on Tributyrin-Agar-Plate

**Figure 1:**
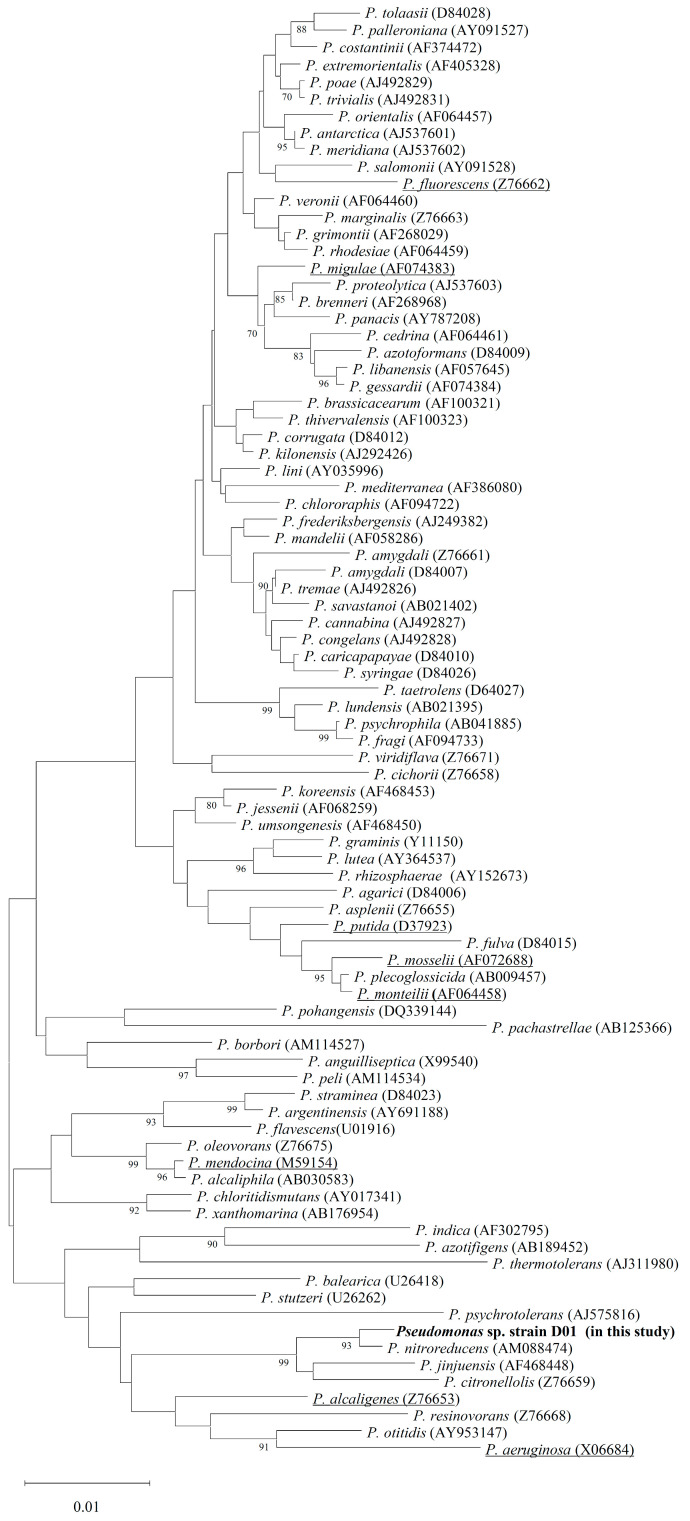
Phylogenetic tree derived from the 16S rDNA of the isolated *Pseudomonas* sp. D01 and 85 *Pseudomonas* representative strains. GenBank accession numbers for sequences are indicated in parentheses. Sequence alignment is performed using Clustal W of MEGA11 software, and the tree is produced by Neighbor-Joining method. Numbers on branches indicate bootstrap value per 1000 replicates. The bar represents the scale of branch length with 0.01 variance in nucleotide sequence. Underlined bacteria are known human pathogenic strains.

We isolated a bacterium from mealworm gut microbiota that grew uniformly on the medium by using the tributyrin minimal-salt (MS) agar plate. 16S rDNA of the isolated bacterium was amplified through polymerase chain reaction (PCR), sequenced, and input into NCBI-Blast. The 16S rDNA between the isolate and *Pseudomonas nitroreducens* DSM14399 (GenBank accession number: AM088474) [28] was found to be 99.35% identical. In support of this, two conserved nucleotide sequences in *Pseudomonas 16S* rDNA, 5′GACGGGTGAGTAATGCCTA3′ and 5′CACTGGTGTTCCTTCCTATA3′ [29], were both found in the ribosomal 16S nucleotide sequences of D01. We retrieved 16S rDNA nucleotide sequences of the other 85 representative bacteria [29] from the NCBI data bank and analyzed their phylogeny. Based on the Neighbor-Joining (NJ) method in MEGA 11 software [30], the isolated bacterium was classified into the genus *Pseudomonas* and designated *Pseudomonas* sp. D01 (Figure 1). According to the phylogenetic tree, *Pseudomonas* sp. D01 is very likely not pathogenic to humans since it is evolutionarily close to nonpathogenic *Pseudomonas* strains, especially the above-mentioned *P. nitroreducens*.

### 2.2. Identification of estD04^+^ Gene from D01 Strain and Classification of EstD04 as an Esterase of bHSL Family IV

**Figure 2:**
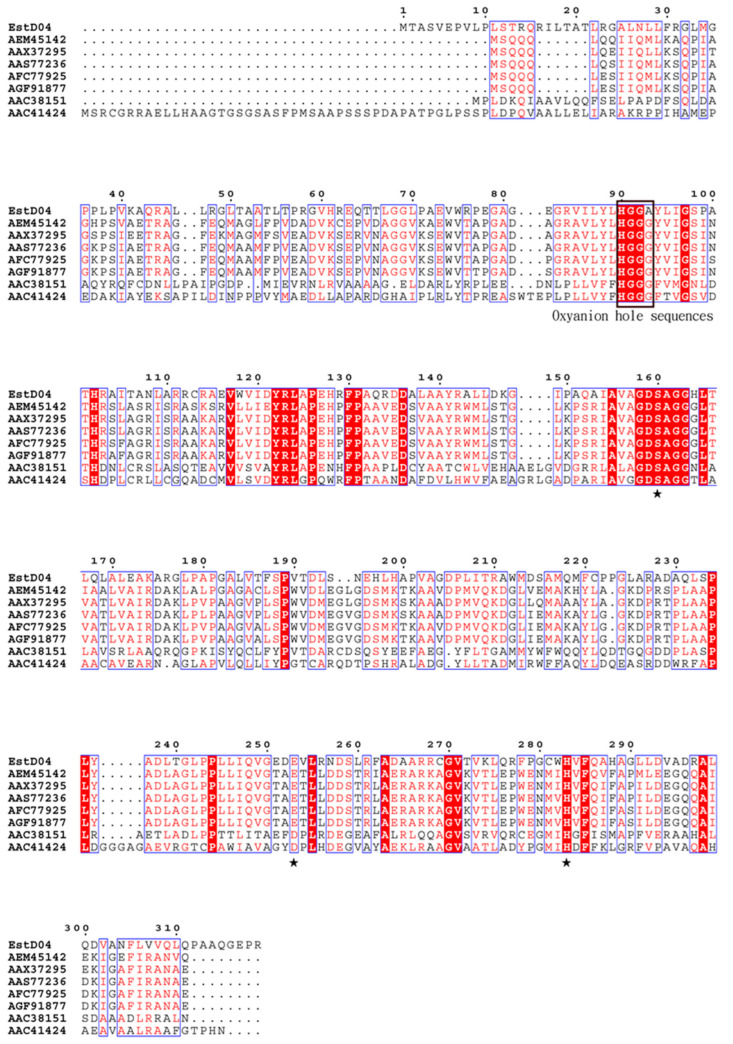
Multiple sequence alignment of EstD04 and its homologs. AEM45142 (Est34, [36]), AAX37295 (α/β hydrolase family 3, 2007), AAS77236 (ELP11B esterase, [37]), AFC77925 (EstC23 esterase [38]), AFG91877 (ArmEst1 esterase [39]), AAC38151 (Lip P enzyme, [40]), and AAC41424 (α/β hydrolase family 3, [41]). Identical residues are highlighted in boxes. Asterisk signals (★) indicate residues constituting the catalytic triad Ser^160^, Glu^253^, and His^283^. Black box indicates the conserved oxyanion hole sequence HGGA^93^.

It was considered that D01 might also contain lipolytic enzymes like *P. nitroreducens* since D01 is evolutionarily close to *P. nitroreducens.* By using in silico search in the Uni-Prot database platform, an α/β-like hydrolase with accession number CEG18-11055 was found in the annotated proteomics of *P. nitroreducens* [31]. We referenced the sequence of *P. nitroreducens* esterase to PCR-amplify the esterase gene on the D01 chromosome and designated it as *estD04^+^*. Based on the analysis from the ExPASY server [32], EstD04 hydrolase has 319 residues with a molecular weight of 34.2 kDa and a pI value of 9.04. However, the signal sequence was not detected in EstD04 using SignalP server 6.0 [33]. NCBI protein Blast analysis indicated that EstD04 is a novel esterase, as the proteins highly similar to EstD04 were all unannotated in NCBI’s GenBank. EstD04 shares only 23~41% of identity with other bHSLs. The most similar one, EstD11 [15], shares only 41% amino acid sequence identity and 38% similarity with EstD04. The catalytic triad residues, the oxyanion hole sequences, and certain conserved amino acids [15,18,34] were identified on EstD04 by amino-acid sequence alignment (Figure 2). 

**Figure 3:**
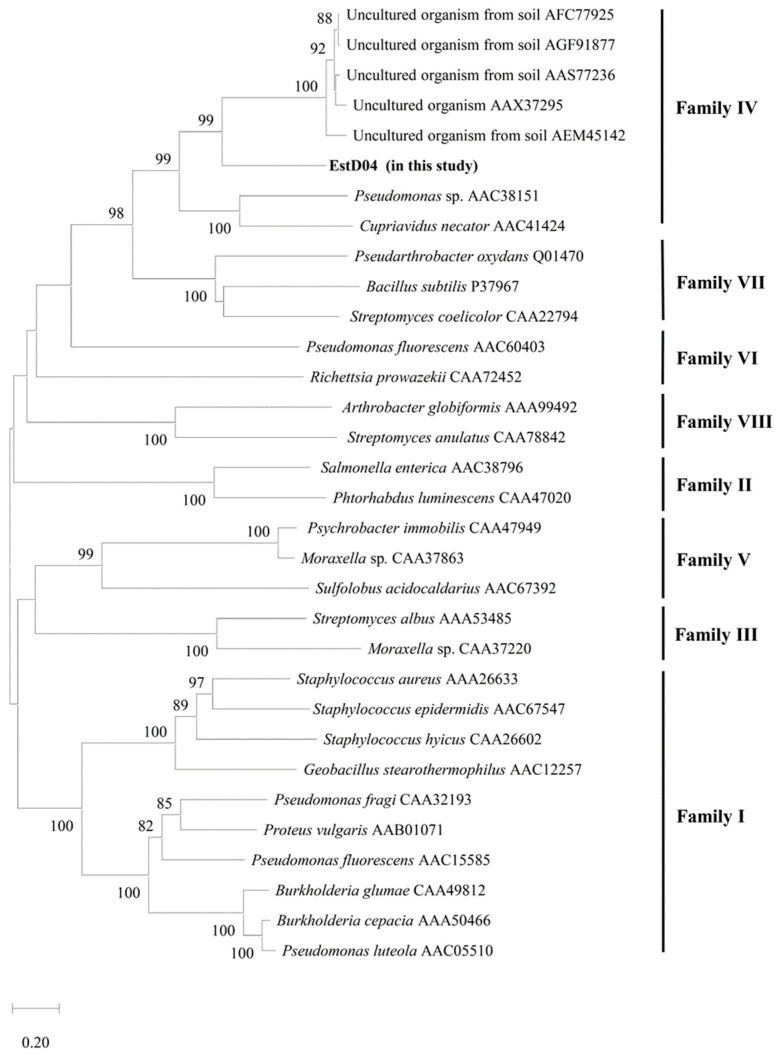
Phylogenetic tree derived from EstD04 esterase protein and 31 representative lipolytic enzymes. GenBank accession numbers of amino-acid sequences are indicated. Sequence alignment is performed using Clustal W of MEGA11 software, and the tree is produced by Neighbor-Joining method. Numbers on branches indicate bootstrap value per 1000 replicates. The bar represents the scale of branch length with 0.20 variance in amino-acid sequences.

**Figure 4:**
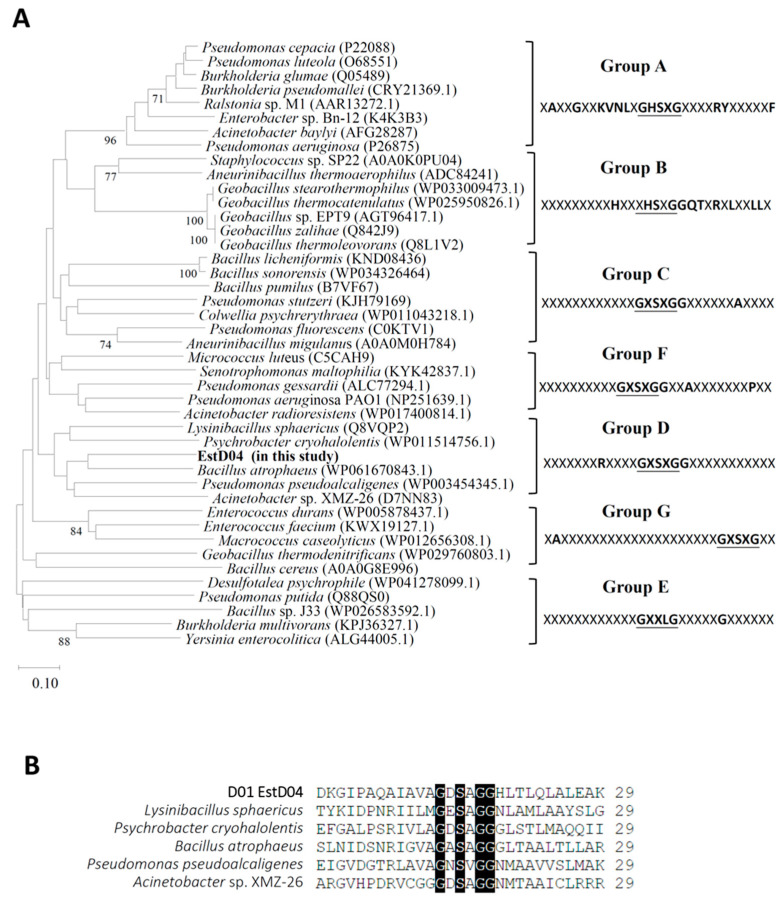
Phylogenetic analysis of the 29 amino acids surrounding the Ser-residue catalytic site of EstD04 esterase and other 42 lipolytic enzymes. (**A**) GenBank accession numbers for amino-acid sequences are indicated in parentheses. Sequence alignment is performed using Clustal W of MEGA11 software, and the tree is produced by Neighbor-Joining method. Numbers on branches indicate bootstrap value per 1000 replicates. The bar represents the scale of branch length with 0.10 variance in amino-acid sequence. Conserved amino acids surrounding the catalytic site are listed for each group. X represents any residue. (**B**) Sequence alignment of the 29 amino acids surrounding the catalytic site of group D (bHSL) members.

Further comparison with 31 full-length lipolytic enzymes retrieved from NCBI [15] revealed that EstD04 belongs to the type IV bHSL family (Figure 3). To clearly demonstrate the phylogeny of EstD04, 29 amino acids that specially surround the catalytic serine residue [8] were retrieved from EstD04 and 42 different bacteria and their phylogeny was analyzed by the NJ method. The results corroborated our finding, suggesting that EstD04 belongs to the group D (bHSL) family amongst the seven lipolytic enzyme groups (Figure 4A). Additionally, in the catalytic region of bacterial esterases, a conserved pentapeptide motif GXSXG is presented, and esterases of the same family tend to have similar motif sequences; for example, type IV esterases usually harbor motifs such as GESAG, GASAG and GNSVG [35]. In comparison, EstD04 also possesses the conserved catalytic triad residues of the type IV esterases (Figure 4B), indicating that it might exhibit biochemical properties and catalytic activities similar to type IV esterases.

### 2.3. Cloning of estD04^+^ Gene and Purification of EstD04-His(6x) Protein

**Figure 5:**
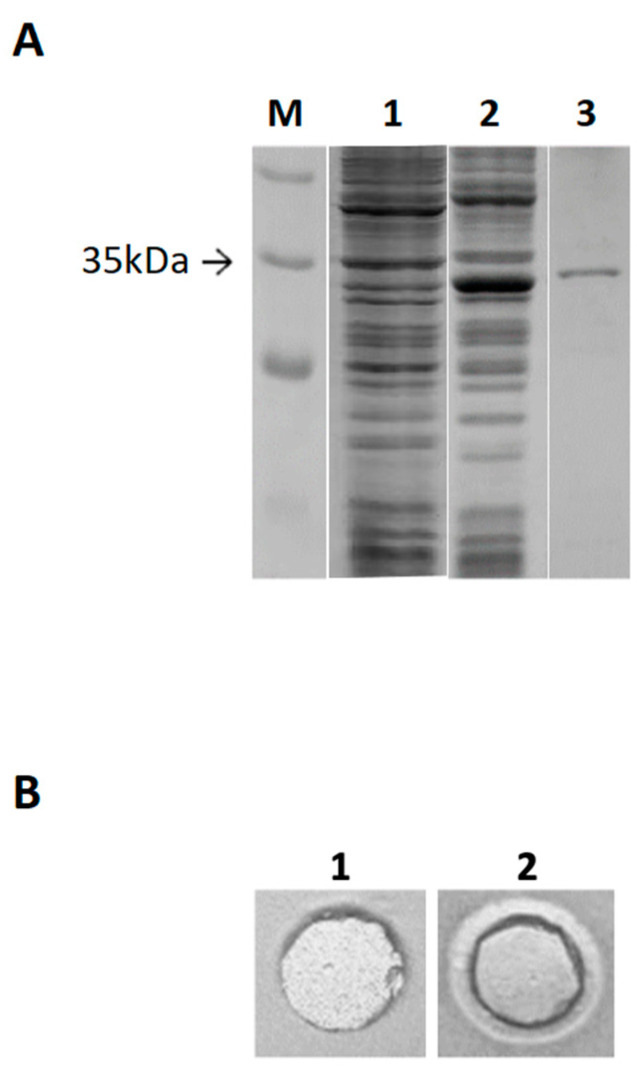
Expression and purification of the recombinant EstD04-His(6x) esterase protein and its lipolytic activity. (**A**) Lane M, protein marker. Lane 1, supernatant from crude lysate of BL21 (DE3) cells harboring plasmid pET21a^+^-*estD04^+^-his(6x)* without IPTG induction. Lane 2, supernatant from BL21 (DE3) cells harboring plasmid pET21a^+^-*estD04^+^-his(6x)* with IPTG induction to produce EstD04 esterase. Lane 3, purified EstD04-His(6x) protein eluted from the Co^2+^ affinity column. (**B**) Formation of halo zone on tributyrin (1%, *v*/*v*) agar plate. Panel 1, control with phosphate-buffered saline (PBS) added. Panel 2, a clear halo zone formed by the purified EstD04 esterase.

To investigate the function of the EstD04 protein, a PCR fragment of *estD04^+^* was attached with a C-terminal his(6x) tag and cloned into pET21a^+^ to express a recombinant EstD04-His(6x) protein. The resultant plasmid pET21a-*estD04^+^*-*his(6x)* was transformed into BL21 (DE3), and EstD04-His(6x) protein was overexpressed by IPTG induction (Figure 5A, lane 1). EstD04-His(6x) enzyme purified by metal affinity chromatography showed only one single band on SDS-PAGE (Figure 5A, lane 2) and formed a clear halo zone on the tributyrin-MS agar plate (Figure 5B, panel 2; panel 1 is a buffer-only control).

### 2.4. Effects of Substrate Chain Length, Temperature, and pH Value on EstD04 Activity and Stability as Well as the Enzyme’s Kinetic Analyses

**Figure 6:**
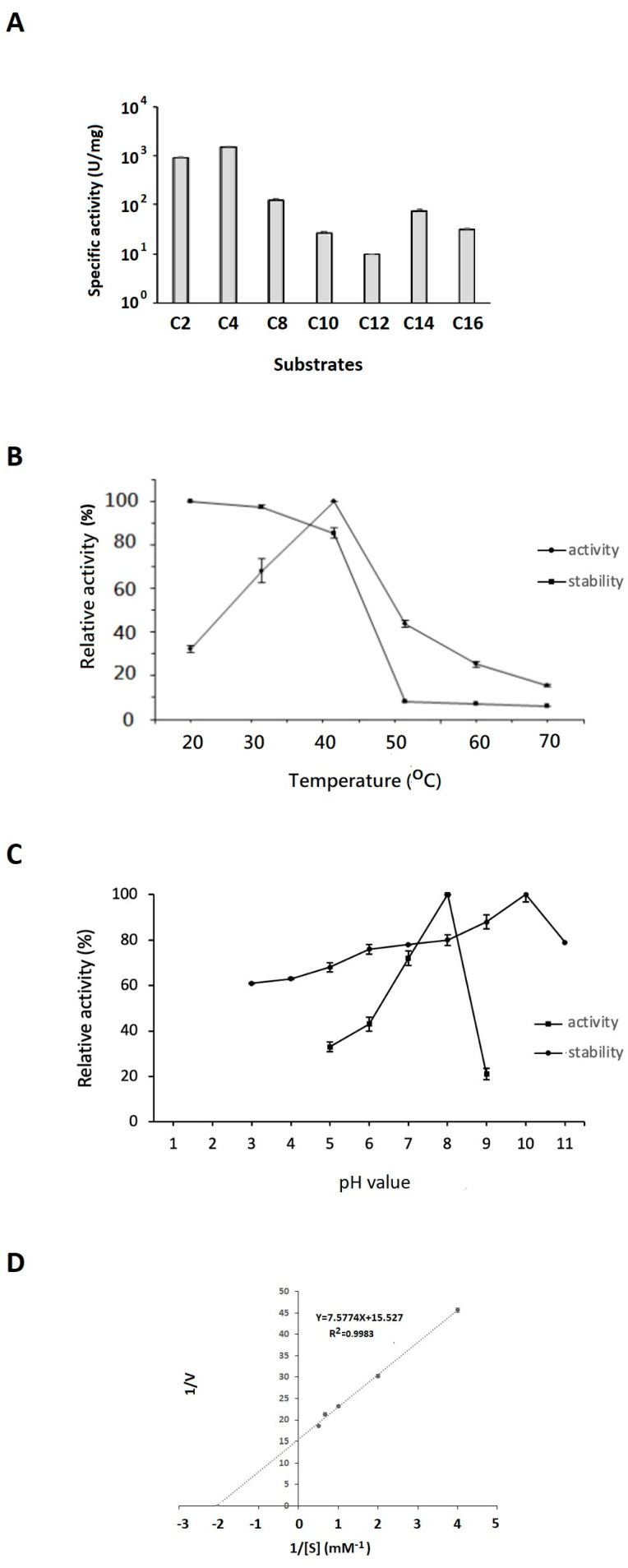
Substrate specificity, effects of temperature and pH on EstD04 enzyme activity, and kinetic analyses. (**A**) Specific activities of EstD04 esterase towards *p*-nitrophenyl esters with different chain lengths, including *p*-nitrophenyl acetate (*p*-NPC_2_), *p*-nitrophenyl butyrate (*p*-NPC_4_), *p*-nitrophenyl octanoate (*p*-NPC_8_), *p*-nitrophenyl decanoate (*p*-NPC_10_), *p*-nitrophenyl laurate (*p*-NPC_12_), *p*-nitrophenyl myristate (*p*-NPC_14_), and *p*-nitrophenyl palmitate (*p*-NPC_16_) at 40 °C and pH 8. (**B**) Specific activities (diamond) and stability (square) of EstD04 under different temperatures. (**C**) Specific activities (square) and stability (diamond) of EstD04 under different pH. The enzymatic activities determined in (**B**,**C**) used *p*-nitrophenyl butyrate (C_4_) as substrate at pH 8 (B) or 40 °C (**C**). Relative enzyme activities were normalized to the highest value. (**D**) Lineweaver–Burk plots of EstD04 for hydrolyzing *p*-NPB. Five substrate concentrations were used: 0.25 mM, 0.5 mM, 1 mM, 1.5 mM, and 2 mM.

The enzyme activity of EstD04 was determined by measuring its hydrolytic capability to *p*-nitrophenyl fatty acids with different carbon chain lengths, including C_2_, C_4_, C_8_, C_10_, C_12_, C_14_, and C_16_. EstD04 exhibited distinct hydrolytic capability on these substrates. As shown in Figure 6A, EstD04 catalytic activity was found to be significantly higher upon hydrolyzing *p*-nitrophenyl C_2_ or C_4_ derivative substrate, whereas noticeable catalytic activity was also observed with *p*-nitrophenyl C_8_, C_10_, C_14_, or C_16_ derivative substrate (Figure 6A). Specifically, EstD04 exhibited the highest catalytic activity towards the C_4_ derivative and the lowest activity towards the C_12_ derivative. These results implied that EstD04 had strong hydrolytic capability for short-chain ester substrates. By using the *p*-NPC_4_ substrate, the specific activity of EstD04 esterase was determined as 1475 U/mg. This is considered to be a high specificity when compared to most other type IV esterases with specific activities ranging from 0.22 U/mg to 426.8 U/mg only [16].

Next, we determined the optimal temperature and pH for the EstD04 esterase function. The different temperatures used to measure the activity of EstD04 were between 20 °C–70 °C. As shown in Figure 6B, EstD04 exhibited optimal enzymatic activity at 40 °C. Moreover, between 20 °C and 40 °C, the enzyme was stable and retained over 82% activity. However, the stability of EstD04, measured by the tolerance of the enzyme to different temperatures, dropped drastically between 50 °C to 70 °C (Figure 6B). The optimal pH for EstD04 activity was pH 8. Finally, we also demonstrated the pH stability (or tolerance) of EstD04 esterase by measuring its enzymatic activity after pre-incubation with buffers of various pH values (Figure 6C). EstD04 exhibited favorable pH stability between pH 8 to pH 11, in which the enzyme retained at least 80% activity. The maximal stability was maintained at pH 10 (Figure 6C), suggesting that this enzyme could be an alkaline lipolytic enzyme. 

By using the optimal conditions for its function, we analyzed the kinetics of EstD04 for the hydrolysis of C_4_ substrates at 40 °C and pH 8. The *K_m_*, *V_max_*, and *k_cat_* values were determined as 0.488 ± 0.001 mM, 64.4 ± 0.9 μMmin^−1^, and 3.01 × 10^3^ ± 40 s^−1^, respectively. The enzyme efficiency, *k_cat_/K_m_* value, was thus calculated to be 6.17 × 10^3^ mM^−1^ s^−1^ (Figure 6D). 

### 2.5. Effects of Cations, Organic Solvents, and Detergents on EstD04 Activity

**Figure 7:**
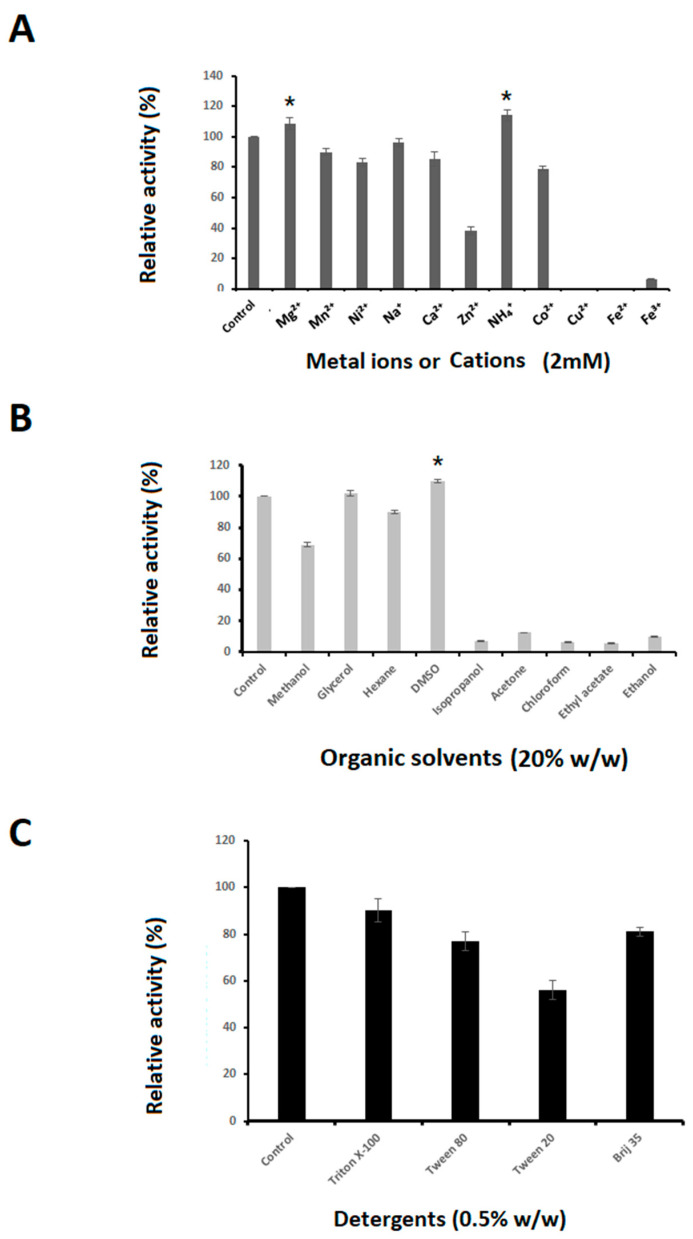
Effect of cations, organic solvents, and surfactants on EstD04 enzymatic activity. The enzymatic activity was determined using *p*-nitrophenyl butyrate (C_4_) as the sample substrate at 40 °C and pH 8.0. (**A**) Enzymatic activities of EstD04 in the presence of 2 mM cations, primary metal ions. (**B**) Enzymatic activities of EstD04 with addition of 20% (*w*/*w*) organic solvents. (**C**) Enzymatic activities of EstD04 with addition of 0.5% (*w*/*w*) surfactants. All enzyme activities are present as relative activity (%) compared to that of the control without addition of cations, organic solvent, or detergent. Error bars: standard deviation of the means. * The relative activity is significantly higher than the control (Student’s t-test, *p*-value < 0.05).

Effects of cations on EstD04 activity are demonstrated in Figure 7A. Compared to the standard determination condition, the enzyme activity of EstD04 increased to about 110% and 115%, respectively, with the addition of Mg^2+^ or NH_4_^+^ to the reaction. EstD04 possessed approximately 80% activity in the presence of metal ions Mn^2+^, Ni^2+^, Na^+^, Ca^2+^, or Co^2+^. On the other hand, it was noticed that Zn^2+^ partially inhibited the catalytic activity of EstD04, and Cu^2+^, Fe^2+^, and Fe^3+^ strongly inhibited the EstD04 enzyme activity (Figure 7A). All of these metal ions were added to the reaction medium at a concentration of 2 mM. Thus, in this study, some of the inhibitory activities were likely elicited by ion toxicity. As reported for lipolytic enzymes of *Pseudomonas* sp. S5, *Burkholderia gladioli* Bsp-1, and *Chryseobacterium polytrichastri* ERMR1:04, metal ions might interact with amino acid side-chain radicals, substantially affecting the ionization of amino acid residues and thereby leading to the enzyme instability [42,43,44].

Investigating the effects of organic solvents and detergents on EstD04 activity, we found that EstD04 esterase retained more than 70% activity when incubated with 20% (*w*/*w*) of methanol, glycerol, hexane, or DMSO when compared to the control (Figure 7B). Notably, it was demonstrated that DMSO increased the esterase activity to nearly 110%. This increased activation might be due to the change in the position of the mobile cap, which could shield the active site: the cap was more flexible in the presence of DMSO, revealing the catalytic center of the enzyme and allowing access to the substrates [45]. Alternatively, it was studied that DMSO could also reduce protein aggregates and increase enzyme solubility by evading hydrophobic interactions within protruding sites on the lipolytic protein structure [46]. EstD04 esterase activity decreased to less than 10% when other organic solvents were used, most likely due to denaturation in the presence of isopropanol, acetone, chloroform, ethyl acetate, and ethanol (Figure 7B).

It is well known that detergents have an amphiphilic structure that can reduce the interfacial tension between oil and water and increase lipid–water interface area, which subsequently enhances the catalytic reaction rates of lipolytic enzymes [47,48]. To demonstrate the effects of detergents on EstD04 esterase activity, different detergents were added individually to the reaction mixture of the enzyme assay. Most detergents [0.5% (*w*/*w*)], including Triton X-100, Tween 80, or Brij 35, had subtle effects on EstD04 enzyme activity (Figure 7C). EstD04 enzyme retained at least 80% activity in each assay. However, Tween 20 had a more deleterious effect on the EstD04 enzyme, which retained only 55% of its activity (Figure 7C).

### 2.6. Molecular Modeling of EstD04 and Ligplot^+^ Analyses

**Figure 8:**
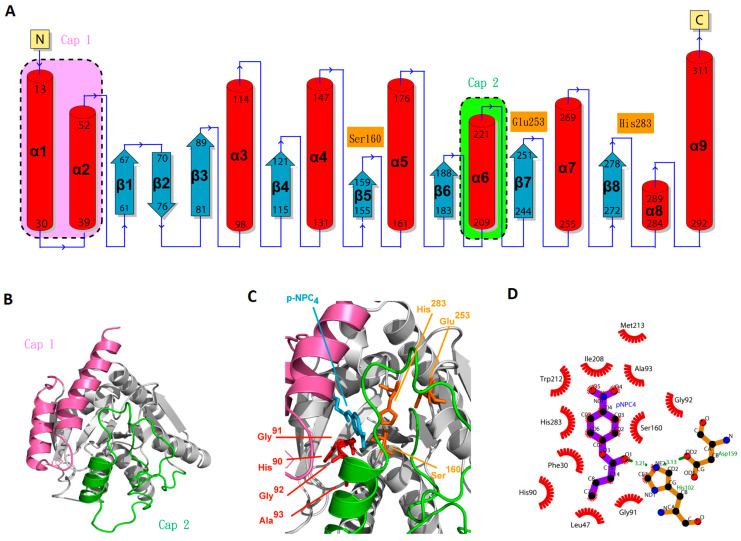
The proposed structures of EstD04 and *p*-NPC_4_-interactive complex. (**A**) Predicted secondary structure of EstD04. (**B**) Monomeric EstD04 with cap subdomain 1 (pink) and cap subdomain 2 (green). (**C**) Complex model of EstD04 with the substrate *p*-NPC_4_. The triad catalytic active site (i.e., Ser^160^, Glu^253^, and His^283^ residues) and a conserved motif, HisGlyGlyAla^93^ (oxyanion hole sequences), are indicated. (**D**) Ligplot^+^ analysis for the protein-substrate interaction of EstD04 and *p*-NPC_4_. The side chains of EstD04 and *p*-NPC_4_ are shown in a ball-and-stick 2D model, with *p*-NPC_4_ colored in purple and amino acid residues colored in orange. Hydrogen bonds are illustrated as green dotted lines, and red spoke arcs represent protein residues that form hydrophobic links with *p*-NPC_4_ atoms. Red balls represent oxygen atoms, black balls represent carbon atoms, and blue balls represent nitrogen atoms.

To find the most propitious template for predicting EstD04 tertiary structure, we selected a 7at0 (EstD11) monomer [15] in the protein data bank (PDB), as it has a match of approximately 41% amino acid sequence identity, 38% similarity, and 90% coverage to EstD04. A tertiary structure of EstD04 was built by using SWISS-MODEL, as shown in Figure 8. As predicted, the secondary structure of EstD04 (Figure 8A) has nine α helices and eight β sheets, typical of an α/β hydrolase. Anyway, the catalytic site is covered by a cap domain consisting of two subdomains. Cap subdomain 1 is located at the N-terminus, including α1, α2, and the hinge site (residues 11–60), and cap subdomain 2 is located around the α6 region (residues 189–240) (Figure 8A,B). To gain insight into the substrate recognition process, we modeled substrate–enzyme interactions by using *p*-NPC_4_ as the sample substrate (Figure 8C). The triad catalytic center of the EstD04 enzyme is constituted by Ser^160^, Glu^253^, and His^283^ residues (Figure 8C). In addition, a conserved motif, HisGlyGlyAla^93^, is located 66 aa(s) away from the catalytic Ser^160^ residue; this motif is near the catalytic serine residue in the tertiary structure and is functionally involved in stabilizing oxyanion hole during the substrate degradation [49] (Figure 8C). Meanwhile, several Proline residues were found surrounding the mobile cap region. Pro^189^ is located in a loop connecting the cap 2 subdomain and the catalytic core [15]. Pro^206^ is located prior to α6 of the cap 2 subdomain. In another loop located at the cap 1 subdomain, Pro^36^ and Pro^57^ were found to function as hinges that stabilize the structure [15,18].

The interaction between *p*-NPC_4_ and EstD04 was further analyzed by using Ligplot^+^ (Figure 8D), characterized by hydrogen bonding and hydrophobic interactions. Ser^160^ and His^283^ in the catalytic triad associated with atoms of *p*-NPC_4_ through hydrophobic interactions. On the other hand, hydrogen bonding (shown as green dotted lines) connects the atoms of *p*-NPC_4_ and His^102^, as well as those of His^102^ and Asp^159^. It was also stated that Asp^159^, a marked motif of the type IV family [15], stabilizes *p*-NPC_4_ through its polar interaction with His^102^. The remaining amino acids mostly render hydrophobic interactions with atoms of *p*-NPC_4_, including the residues in cap 1 (Phe^30^ and Leu^47^), cap 2 (Ile^208^ and Trp^212^), and oxyanion hole (His^90^, Gly^91^, Gly^92^, and Ala^93^). These interaction analyses supported our model of the EstD04 3D structure. 

## 3. Discussion

A novel bacterium capable of using tributyrin as a single carbon source was isolated from the mealworm gut, and its 16S rDNA was PCR-amplified and sequenced. Through phylogenetic analyses of the 16S rDNA, we identified this isolated D01 bacterium as a member of the genus *Pseudomonas*. By using *P. nitroreducens* as a reference model, we identified a novel α/β hydrolase, named EstD04, in silico in our D01 strain and categorized it to the type IV bHSL family. EstD04 presents a distinct sequence, high enzymatic activity, and unique two-cap domain structures. Interestingly, the two cap domains may play a vital role in temperature- or pH-dependent enzymatic activity and enable the enzyme to exhibit distinct physiological characteristics.

In the amino acid sequence comparison, EstD04 is only 23~41% identical to other bHSLs. The most similar one, EstD11 [15], shares only 41% amino acid sequence identity and 38% similarity with EstD04. Notably, when considered a novel enzyme, EstD11 shares 68% identity with the most similar esterase (EstC23). Therefore, EstD04 is very different from other discovered esterases. Moreover, while most of the type IV esterases identified so far are from non-culturable metagenomes, EstD04 was PCR-amplified from the genome of the isolated bacterium D01, and thus EstD04 is one of the few *Pseudomonas* type IV esterases with characterized sequence, enzymatic activity, and bacterium source. Since the D01 strain is evolutionarily close to a nonpathogenic bacterium, *P. nitroreducens*, it may also be nonpathogenic, suggesting that both the enzyme EstD04 and the bacterium D01 are ideal for industrial use.

EstD04 represents an esterase with broad substrate specificity, as it could hydrolyze tributyrin and *p*-nitrophenyl esters with different chain lengths. This is consistent with previous findings that bHSL enzymes, especially those classified in family IV [15,18,27,50], tend to have broad substrate specificity, although they generally favor the hydrolysis of short-chain *p*-nitrophenyl esters and tributyrin [11]. With *p*-NPC_4_ as substrate, we measured and calculated the values of *K_m_* = 0.488 ± 0.001 mM, *k_cat_* = 3.01 × 10^3^ ± 40 s^−1^ and *k_cat_/K_m_* = 6.17 × 10^3^ mM^−1^ s^−1^ for EstD04 catalytic activity. In general, the typical values of *k_cat_/K_m_* in lipolytic enzymes are between 10^4^ to 1 mM^−1^ s^−1^ [43]. Therefore, EstD04 has a relatively efficient *k_cat_/K_m_* turnover rate. Moreover, the low *K_m_* value indicates that EstD04 has a better binding affinity for *p*-NPC_4_. The high activity rate of EstD04 would propound its use in industrial applications.

Through bioinformatic searches and crystal structure simulations, we identified a variety of conserved motifs for type IV lipases in EstD04 esterase. First, an α/β hydrolase domain is localized within the residues 61–188 and 244–311. The catalytic triad center is composed of Ser^160^(S), Glu^253^(E), and His^283^(H) residues that are located separately in conserved sequences GDSAGG (158~163 aa), EXL (253~255 aa), and HVF (283~285 aa) but are closely juxtaposed in the tertiary structure of EstD04. Finally, a HisGlyGlyAla (90~93 aa) motif is located near the catalytic center of the simulated 3D structure, which could be functional to stabilize the oxyanion hole during substrate degradation, much like the conserved HisGlyGlyGly motif in other esterases [49]. 

In the primary sequence analysis, we found that EstD04 has two cap domains, which are the most salient differences compared to EstD11 (Supplementary Figure S1), the esterase most similar to EstD04. Spatially, both the cap 1 region (residues 11–60) and the cap 2 region (residues 189–243) are structurally located at the N-terminus and have distinct sequences. The two cap regions, located in front of the catalytic triad center in the 3D structure, might mediate the cap movement to allow the access of different substrates [15,18]. Interestingly, while EstD04 exhibited properties of an alkaline enzyme, such as having a pI value of 9.04, an optimal catalytic pH of 8, and tolerance to pH 10–11, EstD11 renders the pI = 5.21 and is active between pH 6.5 to pH 8.5 [15]. Also, EstD04 is a mesophilic enzyme, whereas EstD11 is a thermophilic one. These findings suggest that the discrete cap domains may play a vital role in temperature- or pH-dependent enzymatic activity and enable the two evolutionarily close enzymes to exhibit such distinct physiological characteristics. 

In summary, EstD04, a novel bHSL, displays broad substrate specificity, efficient kinetic properties, and higher chemical stability. EstD04 is unique, and not only is its sequence distinct from other esterases, but it is also a rare *Pseudomonas* esterase with a thoroughly characterized sequence and activity. In future studies, the crystal structure of the EstD04 protein could be further investigated for its basic mechanisms of substrate-binding, catalytic activity, and mesophilic stability. Moreover, protein engineering strategies could be adopted to design and modify the enzymes to possess higher activities for biotechnological applications.

## 4. Materials and Methods

### 4.1. Reagents, Enzymes, Plasmids, Strains, and Culture Medium

Chemical reagents were purchased from Sigma-Aldrich (St. Louis, MO, USA) and J.T. Baker (Phillipsburg, NJ, USA). Restrictions enzymes, *Bam*HI and *Eco*RI, and T4 ligase were acquired from TAKARA (Kusatsu, Shiga, Japan). The polymerase chain reaction (PCR) kit and gel purification kit were purchased from New England Biolab (Ipswich, MA, USA). Plasmid pET-21a^+^ was obtained from Novagen (Madison, WI, USA) and plasmid purification kits were obtained from Viogene (New Taipei, Taiwan), respectively. *E. coli* XL1 Blue strain was used as a host for gene cloning, and BL21 (DE3) was used as a host for protein expression. M9-tributyrin MS mediums containing 1 g/L K_2_HPO_4_, 1 g/L KH_2_PO_4_, 1 g/L (NH_4_)_2_SO_4_, 0.1 g/L NaCl, 0.1 g/L NgSO_4_·7H_2_O, 0.04 g/L CaCl_2_, 0.040 g/L FeSO_4_·7H_2_O, 0.01 g/L CuSO_4_, 0.005 g/L MnSO_4_, 0.001 g/L ZnSO_4_, 0.0005 g/L CoCl_2_, 0.005 g/L Na_2_MoO_4_, 1 g/L tween-20, and 1 g/L tributyrin were used.

### 4.2. Isolation and Identification of a Lipolytic Enzyme-Producing Microorganism

Mid guts isolated from mealworms were ground in tributyrin-MS medium, and the medium was poured on tributyrin-MS agar plates. After several days, a bacterium that survived on the plates was isolated. 16S rDNA(s) of the bacterium was amplified by colony PCR using two universal primers, 8F (5′-AGAGTTTGATCCTGGCTCAG-3′) [51] and 1492R (5′-GGTTACCTTGTTACGACTT-3′) [52]. Sequencing of the purified PCR products was performed by Genomics BioSci & Tech (Taipei, Taiwan). The obtained 16S DNA sequence was submitted to GenBank and received accession number OQ306525. Based on previous analysis of *Pseudomonas* 16S rDNA(s) [29] and NCBI-BLAST, the 16S rDNA sequence of 85 *Pseudomonas* bacteria was retrieved from NCBI GenBank. The acquired 16S rDNA sequences were aligned by using the Clustal W program, and their phylogenetic tree was created by the Neighbor-Joining (NJ) method with 1000 bootstrap replicates in the MEGA 11 program. 

### 4.3. Extraction of Pseudomonas sp. D01 Chromosomal DNA

*Pseudomonas* sp. D01 was cultured in LB broth at 37 °C with shaking at 150 rpm for 16 h. Chromosomal DNA was then extracted by using Tissue & Cell Genomic DNA purification kit (GeneMark, Taichung, Taiwan).

### 4.4. Prediction of estD04^+^ Gene and Phylogenetic Analyses of Its Encoding Protein

Referring to the sequence of α/β hydrolase of *Pseudomonas nitroreducens* in the Uni-Prot data bank [31], two PCR primers were designed to base pair the nucleotide sequences located upstream and downstream of the *estD04^+^* putative gene. By using the chromosome of D01 as a template for PCR, a single DNA fragment of the correct size was amplified. After sequencing, the exact nucleotide sequence of the *estD04^+^* gene was determined. The amino acid sequence of EstD04 (a protein encoded by *estD04^+^*) was analyzed by the ExPAsy server [32]. The possible signal sequence of EstD04 was predicted by SignalP server 6.0 (https://services.healthtech.dtu.dk/services/SignalP-6.0, accessed on 1 March 2023). The full-length amino-acid sequence of other lipolytic enzymes, especially those listed in reference [15], were retrieved from the Genbank database and aligned with the sequence of EstD04 by Clustal W. A phylogenetic tree of the protein sequence of EstD04 and 31 other different lipolytic enzymes was constructed by NJ method with 1000 bootstrap replicates in MEGA 11 program [30]. Another phylogenetic tree was constructed with 29 amino-acid residues adjacent to the serine in a catalytic triad from EstD04 and 42 other different bacterial lipolytic enzymes [8].

### 4.5. Cloning, Overexpression, and Purification of the Recombinant EstD04

The *estD04^+^* gene with C-terminal (6x) His nucleotides was PCR-amplified from the chromosome of D01 and ligated into a pET-21a^+^ vector by using restriction enzyme cutting sites *Bam*HI and *Eco*RI. The ligation product was transformed into *E. coli* XL1-Blue and the transformants were selected by LB agar plates with 100 μg/mL ampicillin. The resulting plasmids pET-21a^+^-*estD04*^+^-*his(6x)* were extracted from the transformants, and the one confirmed with the correct nucleotide sequence was transformed into *E. coli* BL21 (DE3) for EstD04 protein expression.

Next, *E. coli* BL21 (DE3) with pET-21a^+^-*estD04*^+^-*his(6x)* were cultured in LB medium with ampicillin (100 μg/mL) to OD600 = 0.4. Isopropyl-β-d-1-thiogalactopyranoside (IPTG) was added to the culture at a concentration of 0.3 mM) and the culture was cultured for a further 4 h at 37 °C. Bacterial cells were collected by centrifugation. The pellets were dissolved in a buffer solution (50 mM NaH_2_PO_4_, 500 mM NaCl, 10 mM imidazole, 10% glycerol at pH 8.0), and the cells were lysed by sonication. After centrifugation at 10,000× *g* for 25 min (min) at 4 °C, the supernatant was collected and filtered through Millex@HA filtration apparatus (Merck Millipore, Burlington, MA, USA) before pouring into Talon ^®^metal affinity resin column (TAKARA, Kusatsu, Shiga, Japan). After several washes, the EstD04 esterase protein was eluted with 200 mM imidazole. An Amicon^®^ Ultra-15 Centrifugal Filter Device (Merck Millipore, Darmstadt, Germany) was used to dialyze and concentrate the purified protein in PBS (1.47 mM KH_2_PO_4_, 137 mM NaCl, 2.68 mM KCl, 8.1 mM Na_2_HPO_4_). The concentrated EstD04 esterase protein was detected by SDS-PAGE with Coomassie Brilliant Blue R-250 staining, and the protein concentration was measured by Bradford’s method [53].

### 4.6. Substrate Specificity Assays

The preferential substrates for EstD04 enzyme were determined by the activity of hydrolyzing various synthetic *p*-nitrophenyl acyl esters, such as *p*-NP acetate (C_2_), *p*-NP butyrate (C_4_), *p*-NP octanoate (C_8_), *p*-NP decanoate (C_10_), *p*-NP dodecanoate (C_12_), *p*-NP myristate (C_14_), *p*-NP palmitate (C_16_), and *p*-NP stearate (C_18_). Each acyl ester was first dissolved in isopropanol as a stock solution (20 mM). A reaction mixture (total volume: 0.8 mL) was prepared with EstD04 (2 ug/mL) and substrate (2 mM) in potassium phosphate buffer (50 mM) at pH 8.0. The reaction mixture was then incubated at 40 °C for 5 min and shifted to 4 °C to terminate the enzymatic reaction. End products from the decomposition of *p*-NP esters were quickly detected by a spectrophotometer at 405 nm. An enzyme-free solution was used as a control, and all the experiments were executed in triplicate. One unit (U) of esterase activity is defined as the amount of enzyme to release 1 µmol of *p*-nitrophenol in 1 min under the assay condition. Relative esterase activity is defined as the percentage of each substrate-specific esterase activity to the activity of consuming the most susceptible substrate in the assay.

### 4.7. Effects of Temperature and pH on EstD04 Enzyme Activity

The optimal temperature for EstD04 esterase activity was determined by assaying the relative activity of the hydrolyzing *p*-NPB (C_4_) substrate at various temperatures (20, 30, 40, 50, 60, and 70 °C). The reaction mixtures were prepared and assayed as described above at different temperatures. The temperature stability (tolerance) of EstD04 was determined by pre-incubating the enzyme at different temperatures (20, 30, 40, 50, 60, and 70 °C) for 5 min before measuring the enzyme activity at pH 8.0 and temperature 40 °C.

The optimal pH for esterase activity was determined by measuring the relative activity of the hydrolyzing *p*-NPB (C_4_) substrate over a pH range of 3–10. The pH value of the reaction mixture was adjusted by using sodium citrate-phosphate (pH 3–6), potassium phosphate (pH 7–8), Tris-HCl (pH 8–9), and glycine-NaOH (pH 10–11), respectively. The pH stability (tolerance) of EstD04 was determined by pre-incubating the diluted enzyme in buffers with pH 5, 6, 7, 8, and 9 at 37 °C for 5 min before assaying, and then enzyme activity was determined at optimum conditions such as pH 8 and 40 °C.

### 4.8. Kinetic Analyses

*p*-NPB (C_4_) substrate was added to reaction mixtures at final concentrations of 0.25 mM, 0.5 mM, 1 mM, 1.5 mM, and 2 mM, separately. The enzyme activities were then measured at 40 °C and pH 8. A Lineweaver–Burk double reciprocal plot was plotted by using Excel 2016 software to present the relationship between substrate concentration and the initial reaction rates. Kinetic constants, *K_m_*, and *V_max_*, of EstD04 were determined by the x-intercept (−1/*K_m_*) and the y-intercept (1/*V_max_*), respectively. Catalytic constants (*k_cat_* and *k_cat_/K_m_*) were determined by dividing *V_max_* with the enzyme concentration [54]. 

### 4.9. Effects of Cations, Organic Solvents, or Surfactants on EstD04 Activity

The effects of cations on esterase activity were determined by measuring the residual activity of the enzyme after incubation with 2 mM of different cations for 30 min. The cations included Na^+^ (NaCl), Mg^2+^ (MgCl), Ca^2+^ (CaCl), Mn^2+^ (MnSO_4_), Fe^2+^ (FeSO_4_), Co^2+^ (CoCl_2_), Cu^2+^ (CuSO_4_), Zn^2+^ (ZnSO_4_), Ni^2+^ (NiSO_4_), Fe^3+^ (FeCl_3_), and NH_4_^+^ (NH_4_Cl). A negative control, without adding any cations, was also included in the assay.

The effects of organic solvents or detergents on EstD04 esterase activity were analyzed in a similar manner. Instead of cations, organic solvents (final concentration: 20% *w*/*w*) or surfactants (final concentration: 0.5% *w*/*w*) were used to incubate with EstD04 enzyme (final concentration: 0.2 μg/mL) in the assays. Organic solvents included methanol, glycerol, hexane, DMSO, isopropanol, acetone, chloroform, ethyl acetate, and ethanol; the detergents included Triton X-100, Tween 80, Tween 20, and Brij35. The relative esterase activity was determined by comparing the enzyme activity to that of a control without solvent or detergent.

### 4.10. Modeling and Protein-Substrate Interaction Analyses

The web-based protein structure prediction software SWISS-MODEL was used to predict the structure of EstD04. Structure 7at0 in the PDB database was chosen as the template for model building due to its better identity, similarity, and coverage of amino acid sequence to EstD04. To build the enzyme–substrate complex model, EstD11-S144A:NP-*p*NP complex structure (PDB no.: 7NB5) was selected as a template. The molecular model or structure of EstD4 and *p*-NPB (C_4_) were superimposed to the template, and the proposed complex model of EstD04- *p*-NPB (C_4_) was generated after energy minimization. To further analyze the detailed contacts within EstD04 and *p*-NPB (C_4_), a complex analysis was performed by LigPlot^+^ according to the software guidance [55].

### 4.11. Statistical Analysis 

The standard deviation of each experimental result was determined by triplicate experiments using triplicate samples. Student’s *t*-test was used to determine the significant difference between the two sets of results, and a *p*-value < 0.05 was considered to be significant.

## 5. Conclusions

From mealworm gut microbiota, a new *Pseudomonas* bacterium D01 that can hydrolyze tributyrin was isolated, and its esterase EstD04 was identified in silico. Recombinant EstD04 expressed and purified from *E. coli* exhibited superior lipolytic activity and broad substrate specificity. The results from bioinformatic analyses and tertiary structure simulation suggested that EstD04 could be a bHSL of the type IV lipase family. Finally, the unique sequence characteristic of EstD04 indicated that its two cap domains may be involved in the mesophilic catalytic function. These findings provide a possible direction for the engineering of lipolytic enzymes and merit further evolutionary or functional studies.

## Data Availability

All data are available in the text of the paper.

## Supplementary Materials

The following supporting information can be downloaded at: https://www.mdpi.com/article/10.3390/molecules28145410/s1, Figure S1: The sequence alignment of the complete amino acids between EstD04 and EstD11.
