# Supplementary material for: Enzymatic Characterization of a Novel HSL Family IV Esterase EstD04 from Pseudomonas sp. D01 in Mealworm Gut Microbiota
Source: Molecules. 2023 Jul 14;28(14):5410. doi: 10.3390/molecules28145410 (PMC10385968; doi:10.3390/molecules28145410)
Supplement: Supplementary file 1 [file molecules-28-05410-s001.zip › molecules-2475620-supplementary.pdf]

Supplementary Materials

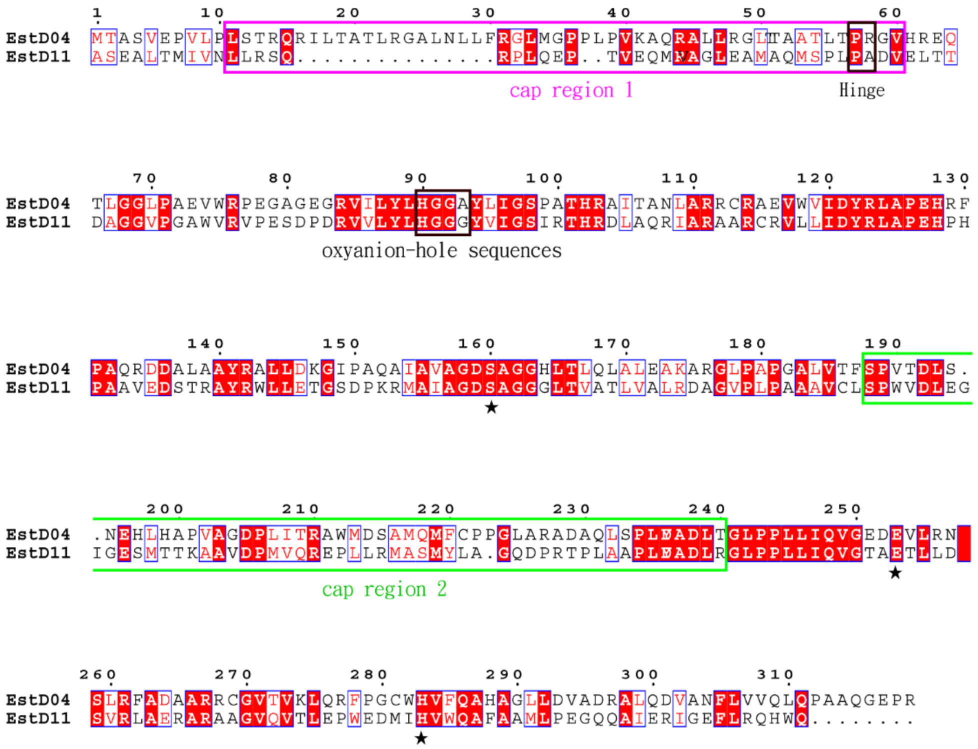

Figure S1. Sequence alignment of EstD04 and EstD11. Identical residues are highlighted in red. Asterisk signals (★) indicate residues constituting the catalytic triad Ser160, Glu253 and His283. Black square indicates the conserved oxyanion-hole sequences HGGA[93]. Cap regions 1 and 2 are highlighted with magenta and lime green squares, respectively.
